# Effects of cognitive-behavioral therapy for insomnia compared with controls among cancer survivors: a systematic review and meta-analysis of randomized trials

**DOI:** 10.1186/s12885-025-14192-y

**Published:** 2025-05-14

**Authors:** Joshua T. Cooper, Ellie Svoboda, Allan V. Prochazka, Duc M. Ha

**Affiliations:** 1 School of Medicine, University of Colorado Anschutz Medical Campus, Aurora, CO USA; 2 Strauss Health Sciences Library, University of Colorado Anschutz Medical Campus, Aurora, CO USA; 3 Division of General Internal Medicine, Department of Medicine, University of Colorado Anschutz Medical Campus, Aurora, CO USA; 4 Division of Pulmonary Sciences and Critical Care Medicine Department of Medicine, University of Colorado Anschutz Medical Campus, Aurora, CO USA; 5 Section of Pulmonary, Critical Care, and Sleep Medicine, Rocky Mountain Regional Veterans Affairs Medical Center, Aurora, CO USA

**Keywords:** Survivorship, Sleep initiation and maintenance, Health-related quality of life, CBT-I, Cancer-related insomnia

## Abstract

**Background:**

Insomnia is highly prevalent among cancer survivors. Meta-analyses examining the effects of cognitive-behavioral therapy for insomnia (CBT-I) among cancer survivors have focused on within-group (pre-to-post-intervention) changes, with calls to better evaluate treatment effects.

**Objective:**

To conduct a systematic-review and meta-analysis and evaluate the effects of cognitive-behavioral therapy for insomnia (CBT-I) among cancer survivors, compared with controls, on insomnia.

**Methods:**

We followed recommendations from the Cochrane Handbook and PRISMA guidelines. We comprehensively searched 8 databases (CINAHL/ClinicalTrials.gov/Cochrane Central/Embase/MEDLINE/PEDro/PsychInfo/Web of Science) and included randomized controlled trials (RCTs) in which adult cancer survivors with clinically-significant insomnia were randomized to CBT-I or control conditions that included usual care, wait-list, attention, or sleep hygiene education only. We designated the primary outcome as end-of-intervention Insomnia Severity Index (ISI) and secondary outcomes included sleep diary parameters, fatigue, and health-related quality of life (HRQL). We analyzed between-group mean differences (MD’s), standardized-mean-differences (SMD’s), and interpreted results using minimal clinically important difference (MCID) thresholds as endorsed by the American College of Physicians (ACP) or SMD thresholds. We rated evidence certainty using GRADE, facilitated by GRADEpro GDT.

**Results:**

We included 19 RCTs involving 1,803 participants. Participant mean age was 55 and time-since-diagnosis was 2.5 years; 94% were women, mostly survivors of breast cancer. At end-of-intervention, compared with controls, CBT-I improved ISI [MD (95% CI): -4.4 (-5.3, -3.5) points; assessed in 13 trials] that did not reach the MCID threshold (i.e., ≥ 6 points) to suggest that many patients derived clinically-important benefit, but is higher than half of the minimal-important-change (MIC) (i.e., 3-<6 points, including 95% CI), suggesting that an appreciable number of patients derived clinically-important benefit. Subjective sleep diary (assessed in 12 trials) sleep latency, wake after sleep onset, sleep efficiency, fatigue (11 trials), and HRQL (10 trials) were also improved; however, on average, none of the improvements reached their respective MCID or SMD thresholds to suggest that many patients derived clinically-important benefits. In pre-specified subgroup analyses, no intervention or cancer-related characteristics meaningfully changed results. Evidence certainty was low-to-very-low, primarily due to heterogeneity, performance, publication, and/or reporting bias.

**Conclusion:**

Compared with controls, CBT-I improved insomnia at an average magnitude greater than half of the MIC but did not reach the MCID threshold, suggesting that an appreciable number, not many, of cancer survivors derived clinically-important benefit. Strategies are needed to improve insomnia for many cancer survivors, particularly among non-responders to first-line CBT-I.

**Protocol registration:**

PROSPERO (CRD42022332584).

**Supplementary Information:**

The online version contains supplementary material available at 10.1186/s12885-025-14192-y.

## Introduction

Insomnia – trouble with falling or staying asleep, or early awakening – is common among cancer survivors (i.e., anyone living with or beyond a cancer diagnosis), with prevalence up to 70% [1, 2] that is thrice compared to the general population [3], varying by age, cancer type, treatment, method of assessment, and diagnostic criteria [4]. Insomnia can refer to insomnia symptom, syndrome or cluster of sleep symptoms, or clinical diagnoses guided by the Diagnostic and Statistical Manual of Mental Disorders (DSM) or International Classification of Sleep Disorders (ICSD) [5, 6]. The etiology of insomnia in cancer is complex and involves inter-relationships between biological, behavioral, physiological, psychological, and cancer treatment-related adverse effects [3]. Insomnia substantially increases daytime fatigue, lead to social and cognitive dysfunction [7], and impair health-related quality of life (HRQL) [8]. Despite its clinical importance, however, insomnia is often underrecognized and inadequately treated [9]. Strategies are needed to improve insomnia, reduce daytime dysfunction, and enhance the HRQL of cancer survivors [9].

Cognitive-behavioral therapy for insomnia (CBT-I) is a multi-component intervention that includes, but is not limited to, a combination of sleep hygiene education, stimulus control, sleep restriction, cognitive restructuring, and relaxation training [10]. CBT-I is the recommended first-line non-pharmacological treatment for patients with insomnia [10] – with established efficacy/effectiveness among individuals with chronic insomnia [11], comorbid psychiatric, and medical conditions including cancer [12] – and effects greater in psychiatric than in medical populations [13]. Among cancer survivors, systematic reviews and meta-analyses evaluating the effects of CBT-I have been limited by a relatively small number of search databases, analyses of within-group changes (i.e., end-of-intervention compared to baseline) [14], or standardized measures of between-group differences [15] to assess treatment effects. Within-group changes from baseline can be erroneous in assessing treatment effects on insomnia due to the natural course of disease, regression to the mean, cancer-related effects, and other non-specific effects, with propensity for biases [16], particular in conditions that are complex and with high potential for disease status changes such as cancer. Standardized measures are helpful to aggregate different measures but do not allow for absolute magnitude assessments to inform clinical meaningfulness; they are not necessary when the same outcome measure is used across randomized controlled trials (RCTs) [e.g., Insomnia Severity Index, (ISI)]. In addition, the choice of comparator group is of important consideration to assess intervention effects – RCTs comparing CBT-I with another intervention suggested or shown to be equally effective in improving insomnia (e.g., physical exercise, mind-body exercise) should be excluded. Finally, prior meta-analyses identified a need for mechanistic insights on CBT-I in cancer, evaluation of the effectiveness of individual and combination of CBT-I components, impact of behavioral vs. cognitive modifications, a need for clinical diversity, and evaluation of important downstream patient-centered outcomes [14, 15].

The primary objective of this project was to conduct a more comprehensive systematic review and meta-analysis to examine the effects of CBT-I, compared with controls, on insomnia among cancer survivors. Secondary objectives included assessment of potential modifying effects related to cancer characteristics, CBT-I components or duration, and relationships between insomnia control with fatigue and HRQL, two important patient-centered outcomes in cancer survivorship care [17].

## Methods

We registered this protocol on PROSPERO (CRD42022332584) and followed recommendations from the Cochrane Handbook for Systematic Reviews of Interventions [18] and the Preferred Reporting Items for Systematic Review and Meta-Analysis guideline [19].

### Search methods

We expanded the literature search from prior systematic reviews of five [14, 15], to eight databases, including three additional databases: Cumulated Index to Nursing and Allied Health Literature (CINAHL), Physiotherapy Evidence Database (PEDro), and Web of Science. The search strategy was designed to capture the concepts of cancer and survivors, CBT, sleep disturbances/insomnia, and RCTs. A health sciences librarian (ES) conducted searches on July 2022, with queries submitted to: MEDLINE ALL (Ovid, 1946-present); Embase (Embase.com, 1974-present); Cochrane Central (Cochrane Library, Wiley); American Psychological Association PsychInfo (Ovid, 1806-present); ClinicalTrials.gov; Nursing and Allied Health (CINAHL, Elton B. Stephens Company, 1981-present); Physiotherapy (PEDro, 1929-present); and Web of Science (E-Table 1). Prior to search, Google Scholar was considered as a potential database but was ultimately excluded as it did not identify additional studies. All records were de-duplicated in EndNote 21, a citation management software; unique citations were uploaded to Covidence, a data management system for systematic reviews (Melbourne, Australia). Reference lists from existing systematic reviews [14, 15] were reviewed to crosscheck studies.

### Target population

We reviewed all unique citations and included full articles and abstracts published in English that involved adult (i.e., age ≥ 18 years) outpatient cancer survivors of breast and other types, at any time along the survivorship life course (i.e., diagnosis to beyond five years), and with clinically-significant levels of insomnia, including by established clinical criteria (DSM; ICSD), validated questionnaires [e.g., Insomnia Severity Index (ISI) ≥ 8 points], or sleep parameters (e.g., sleep diary mean sleep latency > 30 min). We included RCTs in which participants were allocated to receive CBT-I or usual care, attention or wait-list control, or sleep hygiene education only, a component that is ineffective in improving insomnia (akin to placebo) and not recommended by the American Academy of Sleep Medicine (AASM) as a single-component intervention [10]. We excluded studies that enrolled inpatients with cancer, used non-randomized designs, or RCTs in which participants in the comparator group received treatments suggested to be similarly efficacious/effective in improving insomnia (e.g., non-inferiority trials comparing CBT-I with exercise training [20] or acupuncture [21]); or sleep aids / pharmacotherapy), or experimental conditions that do not meet the AASM definition of CBT-I (e.g., hypnosis).

### Intervention

We included CBT-I interventions as defined by the AASM: “[a single- or multi-component intervention consisting of] “sleep hygiene education, stimulus control, sleep restriction therapy, cognitive therapy, relaxation therapy, and other counter-arousal methods,” with relaxation therapy or counter-arousal methods defined as “structured exercises designed to reduce somatic tension (e.g., abdominal breathing; progressive muscle relaxation; autogenic training) and cognitive arousal (e.g., meditation; guided imagery training) that may perpetuate sleep problems” [10]. We additionally characterized interventions as brief behavioral therapy for insomnia (BBT-I) [10] – an abbreviated version of CBT-I that consists of four sessions delivered over four weeks, emphasizing behavioral components [10]. We provided definitions of CBT-I, BBT-I, and individual components in E-Table 2.

### Outcome measures

To enhance study design, we created a directed acyclic graph [22] that hypothesized potential mechanisms of the relationships between cancer, CBT-I, and outcomes, in which cancer diagnosis, treatment, and cancer-related fear / worries can lead to sleep difficulties and insomnia, with CBT-I influencing sleep behaviors / parameters to improve insomnia, that in turn, improves downstream outcomes fatigue and HRQL (E-Figure 1). We followed recommendations for meta-analyses to include outcome concept, specific measurement, metric, method of aggregation, time point [23], and defined the primary outcome as: insomnia as measured by the ISI total score, calculated as the absolute mean difference (MD) compared with controls, at end-of-intervention. The ISI assesses insomnia severity, degree of interference with daily functioning, extent of worry, and impact on HRQL. Total scores range 0–28 points; higher scores indicate worse insomnia. The ISI has excellent internal consistency (Cronbach’s alpha 0.9), adequate discriminant capacity, and convergent validity [24]. The Pittsburg Sleep Quality Index, a common sleep measure, was not considered an appropriate outcome measure as it assesses symptoms of other sleep disorders (e.g., sleep apnea, restless leg syndrome) not expected to be improved with CBT-I. Secondary outcomes were postintervention (end-of-intervention and follow-up): (1) sleep behaviors / parameters [i.e., sleep latency (*minutes*), sleep duration (*minutes*), wake after sleep onset (WASO) (*minutes*), and sleep efficiency (*percent*)], as assessed by sleep diary, actigraphy, or sleep study; (2) fatigue; and (3) HRQL. All sleep behaviors / parameters were converted to their respective units (e.g., hours to minutes), if needed. All outcomes were recorded at baseline, end-of-intervention, and follow-up periods (i.e., 1–3 months; 6 months; 12 months postintervention).

### Study selection, data extraction, and data handling

Two authors (JTC, DMH) independently reviewed studies, first by examining titles and abstracts to code them for relevance, as “include,” “unclear,” or “exclude;” then full-texts coded as “include” or “unclear,” with disagreements resolved by consensus. A decision record was kept and simple agreement with kappa statistics were calculated [25]. Data from full-length articles were extracted using a standardized form created in Covidence that included participant and cancer characteristics, intervention components, duration, and outcomes. Study characteristics included trial pre-registration and pilot designation as described by the authors or with total enrollment of ≤ 40 participants (20/group). Intervention characteristics included session duration (minutes), intervention duration (weeks), specific components used, and mode of delivery (e.g., in-person, telehealth). To align the directionality of fatigue and HRQL measures across studies, where needed, we followed recommendations to reverse the directionality [18], so that higher scores indicate worse fatigue and better HRQL, respectively (E-Table 3). Where numerical values for outcomes were not provided [26, 27], we followed recommendations [18] and visually inspected figures to estimate the outcome means and used SD’s from other trials with similar participant characteristics.

### Risk of bias

Review authors independently evaluated the risk of bias of included studies using the Cochrane Collaboration “Risk of Bias” (RoB) tool [28], broadly similar to a newer version [29]; all disagreements were resolved by consensus. Trials were rated as having “low” (green traffic light), “high” (red), or “some concerns” (yellow) for: selection bias; performance bias; detection of bias; attrition bias; reporting bias; and other potential sources of bias. To assess publication bias, we generated funnel plots of the MD against the standard error (SE) for the ISI and sleep diary sleep behaviors/parameters between groups at baseline, tested using the Egger’s test.

### Data synthesis & statistical analyses

All outcomes were recorded as continuous variables. We recorded the means and standard deviations (SD’s) for each outcome. Where needed, we calculated the SD’s from 95% confidence intervals (CI’s), SE’s, or standardized effect size (Cohen’s d) using standard procedures [18]. All meta-analyses were performed using random effects models. To determine treatment effects, we analyzed the MD’s between CBT-I and control groups at end-of-intervention and follow-up periods for the primary outcome ISI and secondary outcomes sleep behaviors/parameters. For fatigue and HRQL outcomes, we analyzed the standardized mean differences (SMD’s), as Hedge’s g, due to variations in measures used.

We summarized treatment effects as MD’s (and 95% CI’s) and interpreted results using minimal clinically important difference (MCID) thresholds between groups, or where different measures were used, SMD’s. We followed recent calls to analyze and interpret results using between-group MD’s/MCID’s [16], instead of the within-group minimal important change (MIC) thresholds, or statistical approaches (e.g., SMD) where both the MD/MCID and SMD approaches were possible [18]. The MCID refers to the smallest difference between two groups that is considered clinically important and is recommended to assess treatment effect, while the MIC refers to the smallest change within a group [30] that is considered important but is subjected to biases related to other effects such as natural history of disease, regression to the mean, or non-specific effects [16] that can include placebo effects [31] attributed to participant enthusiasm, attention, and expectations [32]. In the cancer context, cancer-related effects such as recurrence and changes in treatments can also confound effects [3]. Therefore, between-group MD’s and interpretation using the MCID’s are methodologically more rigorous and clinically relevant [16, 18, 30].

The MIC (within-group) threshold for the ISI among individuals with chronic insomnia is a 6-point change from baseline [33], derived from an RCT evaluating pharmacologic treatment for adults with primary insomnia [34]. To derive a between-group MCID threshold, we used the approach described by Johnston and colleagues [35] that is also endorsed by the American College of Physicians (ACP) guideline on insomnia [36]: between-group MD/MCID greater than or equal to the MIC (i.e., ≥ 6 points) suggest that many patients gain clinically-important benefit; MD’s up to half of the MIC (i.e., 3-<6 points) suggest that an appreciable number of patients derive clinically-important benefit, and MDs less than half of the MIC (i.e., < 3 points) indicate a small effect and that patients generally do not derive clinically-important benefit. In addition, we used MD/MCID thresholds for sleep parameters as endorsed by the AASM for the pharmacologic treatment of insomnia [37]: objectively-measured sleep behaviors / parameters (by actigraphy or sleep study) sleep latency (≥ 10-minute reduction), sleep duration (≥ 20-minute increase), WASO (≥ 20-minute reduction), and sleep efficiency (≥ 5-percent increase); and subjectively-measured (sleep diary) sleep behaviors/parameters, respectively: ≥20-minute reduction, ≥ 30-minute increase, ≥ 30-minute reduction, and ≥ 10-percent increase. We used SMD thresholds of 0-0.29, 0.30–0.59, and ≥ 0.60 to indicate statistically small, moderate, and large effect sizes, respectively [38], and a threshold endorsed by the AASM to suggest clinical meaningfulness (i.e., ≥ 0.50, moderate effect size) [37].

We assessed statistical heterogeneity across studies using I^2^ statistics and considered thresholds of < 40% as not important; 40–60% moderate; and > 60–90% substantial heterogeneity [18]. To investigate moderate-to-substantial heterogeneity, we conducted a priori subgroup analyses by cancer type, time since cancer diagnosis, cancer treatment completion status, CBT-I duration, delivery mode, and phase of RCT (pilot vs. larger RCTs). We interpreted heterogeneity ≥ 70%, particularly with ≤ 7 RCTs, as unreliable [39]. We conducted sensitivity analyses omitting trials in which mean ISI scores were estimated from figures [26, 27] or a trial in which the intervention used a meditation approach that overlaps with the AASM definition of relaxation training or other counter-arousal methods but not traditionally considered CBT-I [40]. Additional sensitivity analyses used the SMD approach to compare with existing literature.

We conducted all analyses using RevMan Web (8.1.1), IBM SPSS (29.0.2.0), and defined statistical significance as *p* < 0.05. We generated a Summary of Findings table using the GRADE approach that rated the certainty of evidence: on study design, risk of bias, inconsistency, indirectness, imprecision, and other considerations, facilitated by GRADEpro GDT (McMaster University, Canada).

## Results

**Fig. 1:**
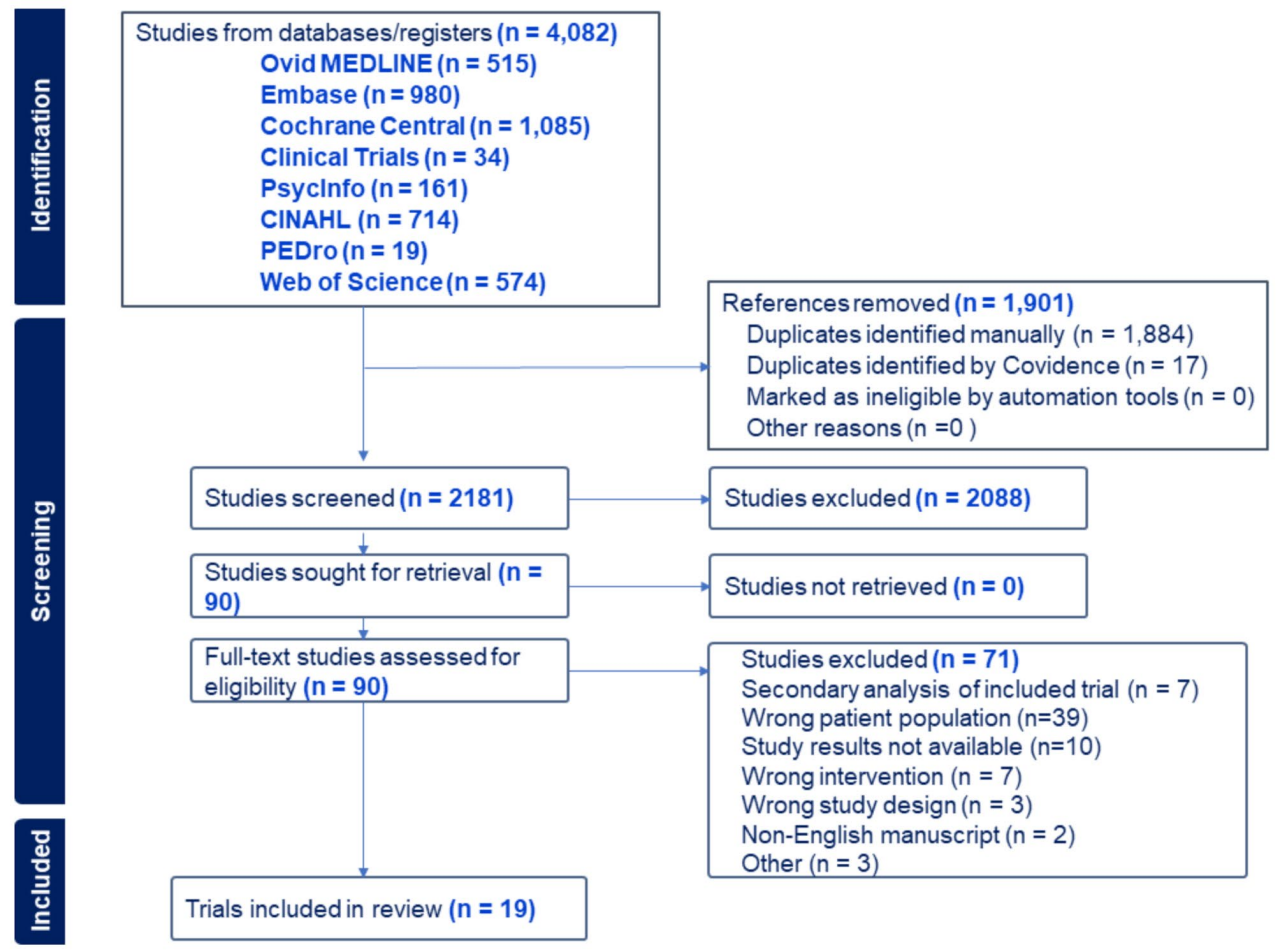
CONSORT diagram CINAHL = Cumulated Index to Nursing and Allied Health Literature; PEDro = Physiotherapy Evidence Database

The initial search resulted 4,082 citations. After de-duplication, 2,181 unique citations were included for title and abstract screening, 90 of which underwent full-text review. There were 26 manuscripts published from 19 RCTs, involving a total of 1,803 participants. Agreement rates between reviewers were 95% (κ = 0.54; moderate) and 83% (κ = 0.58) for the title and abstract, and full-text reviews, respectively. Compared to the latest meta-analysis, of studies up to August 2020 [15], our updated and expanded search, which included two additional years of literature and three additional databases, identified 3,476 more records, resulted in 1,646 more studies screened, 42 more full-texts reviewed, 8 more RCTs (2 of which were published before 2020; 6 published 2020–2022), and an additional 525 participants (Fig. 1).

Of the 19 RCTs, 15 (80%) enrolled exclusively or predominantly (> 50%) survivors of breast cancer. In all, 94% of participants were women, with an average mean (and range) age of 55 (42 to 66) years and time since cancer diagnosis 2.5 years (2 months to 8 years) (Table 1). The average mean (and range) duration of CBT-I was 6 (1–16) total hours, delivered over 6 (4–10) weeks; 15 (80%) of the interventions included a component of live-interaction with an interventionist or therapist. Six (32%) interventions used a hybrid (in-person and remote) delivery format, seven (37%) remote only, and six (32%) in-person only (Table 2).

**Table 1:** Summary of participant characteristics by cancer type

First author, publication year	*N* randomized	Mean age (years)	Women (%)	White race (%)	Sleep inclusion criteria	Predominant cancer type^a^	Cancer stage (%)^c^	Mean time since diagnosis (months, unless specified)	Mean years since treatment completion
Palesh, 2018 [26]	71	51	100	97	ISI	Breast^b^	I (20), II (55), III (23)	3	NR
Savard, 2016 [49]	242	55	100	NR	ISI	Breast^b^	I (52), II (28), III (16)	10	NR
Savard, 2014 [50]	242	53	100	NR	ISI	Breast^b^	I (49), II (34), III (13)	11	NR
Casault, 2015 [51]	38	57	95	NR	ISI	Breast	I-IV (specific stage not reported)	1.8 years	NR
Savard, 2005 [52]	58	55	100	100	DSM-IV	Breast^b^	I (58), II (37), III (5)	3.5 years	2.5
Dirksen, 2008 [53]	81	57	100	94	DSM-IV	Breast^b^	I (50), II (29), III (13)	4.8 years	4.8
Hall, 2022 [27]	40	51	90	90	DSM-IV, ISI	Breast	NR	7.8 years	5.1
Zhao, 2020 [54]	136	53	100	0	AASM	Breast^b^	I (26), II (54), III (20)	NR	2.3
Zachariae, 2018 [55]	255	53	100	NR	PSQI	Breast^b^	I (26), II (46), III (27)	NR	2.9
Ritterband, 2012 [56]	28	54	100	92	DSM-IV	Breast	I (46), II (21), III (18), IV (4)	NR	3.9
Roscoe, 2015 [57]	96	59	88	96	Self-reported/selected	Breast	NR	NR	4.5
Gonzalez, 2022 [58]	30	57	100	100	ISI	Breast^b^	NR	NR	½ to < 5, 1/2 to > 5
Espie, 2010 [59]	150	NR	69	NR	DSM-IV, PSQI	Breast	NR	NR	NR
Matthews, 2014 [60]	60	52	100	NR	ISI, SOL, WASO	Breast^b^	I (36), II (36), III (29)	NR	NR
Palesh, 2020 [61]	74	51	100	62	ISI, SDEF	Breast^b^	I (11), II (40), III (20), IV (26)	NR	N/A
Dean, 2020 [41]	40	66	63	81	ISI	Lung^b^	I (80), II (17)	8	NR
Padron, 2019 [62]	35	59	100	83	WASO, SOL	Gynecologic^b^	I (57), II (9), III (26), IV (3)	N/A	N/A
Zhang, 2019 [40]	70	58	100	0	WASO, SOL, SLE	Cervical^b^	I (66), II (20), III (14)	3.5 years	NR
Chung, 2022 [63]	57	42	81	0	PSQI	Multiple	I (35), II (16), III (35), IV (8)	NR	NR
**Summary**	Total 1,803 Mean (range): 95 (28–255) participants	Mean (range): 55 (42–59) years	Mean (range): 94 (69–100) % women	Mean (range): 59 (0-100)% White race	ISI – 9 studies; DSM – 5 studies; PSQI – 3 studies; Other – 3 studies	15 breast or predominantly breast; 1 lung; 2 gynecologic, 1 mixed	Stage I-II: 20–80% Stage III-IV: 3–35%	Mean (range): 2.7 years (3 months – 7.8 years)	Mean (range): 3.7 (2.3–5.1) years

^a^“Predominant” defined as > 50% of participants having the specified cancer type

^b^100% of participants having the specified cancer type

^c^Percentages do not necessarily add up to 100%, as some studies had participants with unknown or unreported cancer stage

AASM = American Academy of Sleep Medicine; DSM-IV = Diagnostic & Statistical Manual of Mental Disorders, 4th edition; ISI = Insomnia Severity Index; NR = not reported; PSQI = Pittsburgh Sleep Quality Index; SDEF = Sleep Disruption Evaluation Form; SLE = Sleep Efficiency; SOL = Sleep Onset Latency; WASO = Wake After Sleep Onset

**Table 2:** Trial and intervention characteristics

First author, year	Intervention, format	Duration, Weeks (total hours)	One-on- one interaction	Sleep outcomes	Pilot trial	Assessed adherence (Y/*N*) (%)	Assessed intervention fidelity (Y/*N*) (%)
Palesh, 2018 [26]	BBT-CI, Hybrid	4 (3)	Yes	ISI	Yes	Yes (≥ 70)	Yes (≥ 70)
Savard, 2016 [49]	CBT-I, Remote	6 (5)	Optional	ISI, sleep diary	No	Yes (≥ 70)	Yes (≥ 70)
Savard, 2014 [50]	CBT-I, In-person	6 (5)	Yes	ISI, sleep diary	No	Yes (≥ 70)	Yes (≥ 70)
Casault, 2015 [51]	CBT-I, Remote	6 (1)	Yes	ISI, sleep diary	Yes	Yes (≥ 70)	No
Savard, 2005 [52]	CBT-I, In-person	8 (12)	Group	ISI, sleep diary, sleep study	No	No	No
Dirksen, 2008 [53]	CBT-I, Hybrid	6 (5.5)	Mixed	ISI, sleep diary, actigraphy	No	No	No
Hall, 2022 [27]	CBT-I, Remote	4 (3.75)	Yes	ISI, sleep diary	Yes	Yes (≥ 70)	Yes (≥ 70)
Zhao, 2020 [54]	Meditation-focused^*a*^, In-person	6 (9)	Group	ISI, actigraphy	No	No	Yes (≥ 70)
Zachariae, 2018 [55]	CBT-I, Remote	6–9 (4.5-6)	No	ISI, sleep diary	No	Yes (68)	N/A (self-help)
Ritterband, 2012 [56]	CBT-I, Remote	9 (4.5-6)	No	ISI, sleep diary	Yes	Yes (≥ 70)	N/A (self-help/internet)
Roscoe, 2015 [57]	CBT-I, Hybrid	7 (2.5-5)	Yes	ISI	No	Yes (≥ 70)	No
Gonzalez, 2022 [58]	CBT-I, Remote	6 (9)	Group	ISI	Yes	Yes (≥ 70)	Yes (≥ 70)
Espie, 2010 [59]	CBT-I, In-person	5 (4.2)	Group	Sleep diary, actigraphy	No	Yes (≥ 70)	Yes (NR)
Matthews, 2014 [60]	CBT-I, Hybrid	6 (2.5–4.7)	Yes	ISI, sleep diary	No	No	Yes (NR)
Palesh, 2020 [61]	BBT-CI, Hybrid	6 (3)	Yes	ISI	Yes	No	Yes (NR)
Dean, 2020 [41]	BBT-I, Hybrid	4 (2.6)	Yes	ISI, sleep diary	Yes	Yes (≥ 70)	Yes (NR)
Padron, 2019 [62]	CBT-I., In-person	6 (9)	Yes	Sleep diary	Yes	Yes (≥ 70)	Yes (≥ 70)
Zhang, 2019 [40]	Meditation-focused^*a*^, In-person	8 (16)	Group	ISI, sleep diary, actigraphy, sleep study	No	No	No
Chung, 2022 [63]	CBT-I, Remote	10 (8–12)	No	None^*b*^	Yes	Yes (≥ 70)	N/A
Summary	CBT-I (14) BBT-I (3) Meditation- focused^*a*^ (2) In-person (6) Remote (7) Hybrid (6 trials)	Interventions ≤ 6 wks: 13 trials (68%); mean (range): 6 (1–16) total hours	Yes – 9 Group – 5 Mixed – 1 Optional – 1 No – 3 trials	ISI – 16 Sleep diary – 13 Actigraphy – 4 Sleep study – 2 trials	Yes – 9 No – 10 trials	Adherence assessed and ≥ 70% – 12; assessed and < 70% – 1; not assessed – 6 trials	Fidelity assessed and ≥ 70% – 7; assessed but NR – 4; not assessed – 8 trials

^a^Meditation-focused interventions that had overlapping features with CBT-I: relaxation training, cognitive therapy, and other counter-arousal methods (E-Table 2)

^b^Included for secondary outcome HRQL

AASM = American Academy of Sleep Medicine; BBT-CI = Brief Behavioral Therapy for Cancer-related Insomnia; BBT-I = Brief Behavioral Therapy for Insomnia; CBT-I = Cognitive Behavioral Therapy for Insomnia; HRQL = health-related quality of life; ISI = Insomnia Severity Index; NR = Not Reported

Sixteen trials (84%) evaluated 3–4 CBT-I components, two trials [26, 41] evaluated BBT-I, and one trial evaluated a single-component relaxation therapy/meditation approach (E-Table 4). Sixteen trials (84%) assessed the ISI. Sleep parameters, fatigue, and HRQL were assessed in 14 (74%), 11 (58%), and 11 (58%) trials, respectively. Eight trials (42%) assessed participant adherence and therapist fidelity; all trials except for one reported completion rates > 70% [26]. Ten trials (53%) were pre-registered, one retrospectively registered [40], and nine (47%) designated as pilot trials (Table 2). A minority (< 50%) of trials assessed the primary and secondary outcomes beyond end-of-intervention.

All 19 RCTs had at least one domain with high concern for risk of bias – on performance due to lack of blinding of participants and personnel (i.e., participants and/or study personnel were aware of group assignment) or selection (due to inadequate allocation concealment). Approximately 50% of trials did not provide details on blinding of outcome assessment and therefore were rated as with some concern for detection bias. All RCTs had low-to-some concerns on random sequence generation or incomplete outcome data (E-Figure 2). There was evidence suggestive of publication bias, as detected by the primary outcome ISI and secondary outcome sleep diary sleep efficiency, but not sleep latency, duration, or WASO (E-Figure 3).

### Effect of CBT-I on the primary outcome ISI at end-of-intervention

**Fig. 2:**
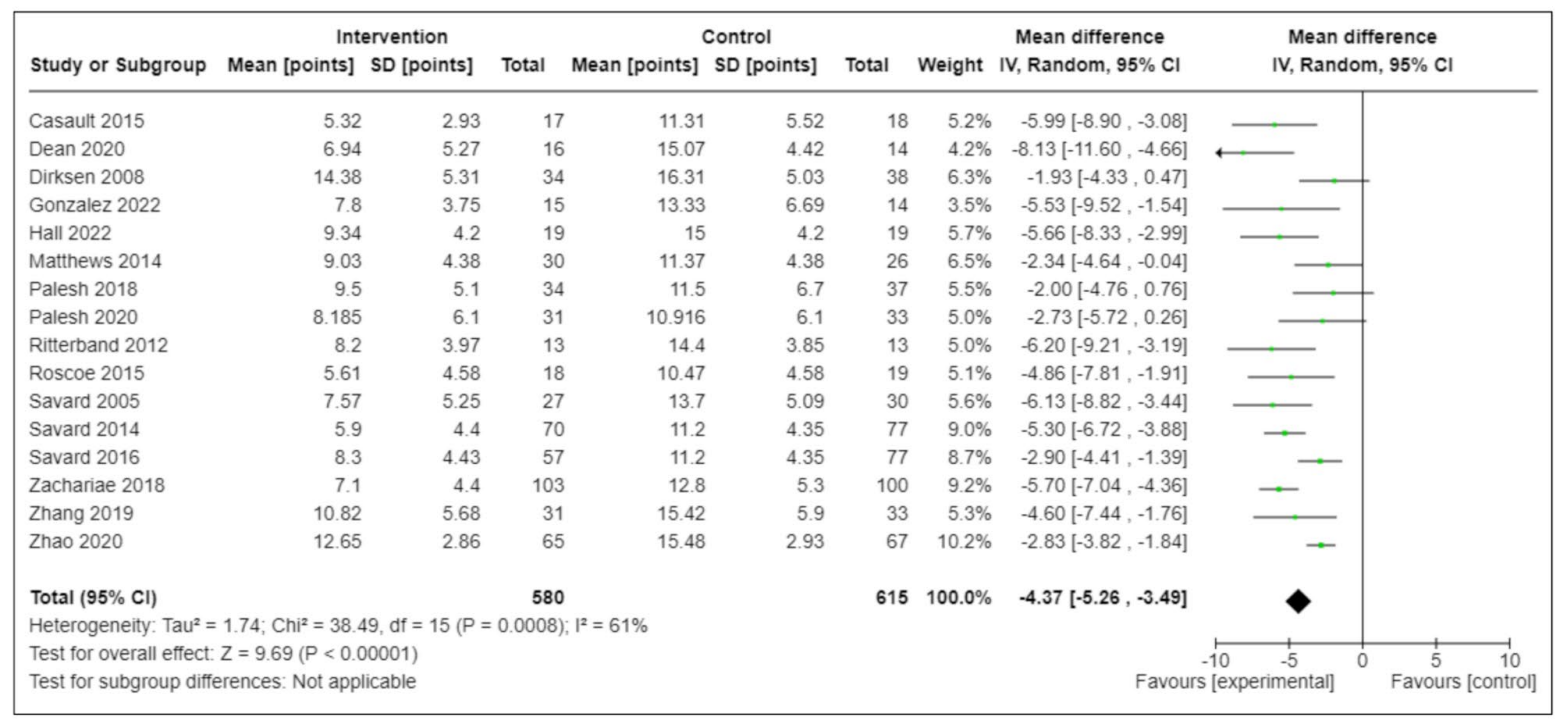
Between-Group Differences in End-of-Intervention ISI (Primary Outcome) CI = confidence interval; ISI = Insomnia Severity Index; SD = standard deviation

Among 1,195 participants across 16 RCTs, ISI scores were similar between groups at baseline. At end-of-intervention, compared with controls, CBT-I participants reported improved ISI, with absolute mean (95% CI) ISI reduction (MD) of -4.4 (-5.3, -3.5) points (I^2^ = 61%) that did not reach the MCID threshold of ≥ 6 points to suggest that many participants derived clinically-important benefit, including the 95% CI (low-certainty evidence, Table 3). The MD was more than half the MIC (i.e., 3-<6 points), however, suggesting that an appreciable number of participants derived clinically-important benefit (low-certainty) (Fig. 2).

**Table 3:** Summary of findings on the effects of CBT-I on End-of-Intervention primary and secondary outcomes

Outcomes	Absolute Effects (95% CI)	№ of participants (studies)	Certainty of Evidence (GRADE)	Comments
ISI	MD **4.4 points lower** (5.3 lower to 3.5 lower)	1195 (16 RCTs)	⨁⨁◯◯ Low^a, b^	On average, did not reach the MCID threshold of ≥ 6 points [24, 33], but reached half of the MIC, suggesting an appreciable number, not many, of participants derived clinically-important benefit.
Fatigue	SMD **0.29 lower** (0.43 lower to 0.15 lower)	831 (11 RCTs)	⨁⨁◯◯ Low^c, d,e^	Statistically small effect size of unclear or no clinical meaningfulness (i.e., on average, did not reach the recommended SMD threshold ≥ 0.50 to suggest clinical meaningfulness) [36, 37].
HRQL	SMD **0.2 higher** (0.04 higher to 0.36 higher)	626 (10 RCTs)	⨁◯◯◯ Very low^d, e^	Small effect size of unclear or no clinical meaningfulness (i.e., on average, did not reach the recommended SMD threshold ≥ 0.50 to suggest clinical meaningfulness) [36, 37].
Sleep Diary Sleep Latency	MD **11.5 min lower** (15.1 lower to 7.9 lower)	936 (12 RCTs)	⨁⨁◯◯ Low [64]^,f^	On average, did not reach the MCID threshold of 20 min [36, 37], but reached half of the MIC, suggesting an appreciable number, not many, of participants derived clinically-important benefit.
Sleep Diary Sleep Duration	MD **4.5 min higher** (6.9 lower to 15.9 higher)	974 (12 RCTs)	⨁◯◯◯ Very low^a, f,g^	Not statistically significant and did not reach the MCID threshold of 30 min [36, 37] nor half of the MIC to suggest clinical-important benefit.
Sleep Diary WASO	MD minutes **14.7 lower** (20.8 lower to 8.5 lower)	937 (12 RCTs)	⨁⨁◯◯ Low^a, f^	On average, did not reach the MCID threshold (including the 95% CI) of 30 min [36, 37], but reached close to half of the MIC, suggesting an appreciable number, not many, of participants derived clinically-important benefit.
Sleep Diary Sleep Efficiency	MD **7.0% higher** (5.2 higher to 8.7 higher)	994 (13 RCTs)	⨁◯◯◯ Very low^a, b,f^	On average, did not reach the MCID threshold (including the 95% CI) of 10% [36, 37], but reached half of the MIC, suggesting that an appreciable number, not many, of participants derived clinically-important benefit.

a. Due to concern for moderate (30–60%) to substantial (50–90%) statistical heterogeneity (by I-squared test), related to study clinical and/or methodological diversity

b. As assessed by funnel plot asymmetry and Egger’s test

c. In addition to concerns on non-reporting or publication bias as suspected with the ISI, fatigue and HRQL outcomes were assessed among only < 60% of included trials

d. Fatigue and HRQL among cancer survivors could be due to other factors other than insomnia and therefore not directly influenced by CBT-I

e. Due to wide confidence intervals

f. Due to absence of established reliability, validity, and responsiveness, and demonstrated large differences (or poor agreement) between sleep diary compared to objectively-measured actigraphy and polysomnography (the gold-standard) parameters (specifically on sleep duration, WASO, and sleep efficiency)

g. Due to wide confidence intervals and directionality of effects (both decreased and increased sleep duration following CBT-I)

CBT-I = cognitive behavioral therapy for insomnia; HRQL = health-related quality of life; ISI = Insomnia Severity Index; MCID = minimal clinically important difference; MIC = minimal important change; MD = mean difference; RCT = randomized controlled trial; WASO = Wake after sleep onset

Subgroup analyses of RCTs that enrolled cancer survivors within 12 months of diagnosis, participants who have completed all cancer treatment, used different CBT-I delivery modes (in-person, remote, hybrid), duration (≤ 4 vs. > 4 weeks or ≤ 6 vs. > 6 weeks), and pilot trials (vs. larger/efficacy RCTs) showed similar magnitudes in ISI reductions at end-of-intervention (MD’s -3.0 to -5.5 points) across subgroups that again, did not reach the MCID threshold but reached half of the MIC, suggesting that an appreciable number, not many, of participants derived clinically-important benefit. Survivors of non-breast cancers might have derived higher ISI reduction [MD: -6.2 (-9.7, -2.8) points], however limited by a small number of two trials (*N* = 94; I^2^ = 58%). There was moderate-to-substantial heterogeneity (I^2^ = 58–61%), with no subgroup substantially reducing (i.e., changed from moderate-to-substantial to not important) heterogeneity, except for trials that used ≤ 6 weeks of CBT-I (6 RCTs; *N* = 560; I^2^ = 0%). There was no evidence of differential treatment effects among subgroups, except for higher ISI reduction for interventions that lasted > 6 weeks [MD: -5.5 (-6.9, -4.1)] compared to those ≤ 6 weeks [MD: -2.9 (-3.6, -2.3) points].

In sensitivity analyses that omitted RCTs in which the mean ISI scores were estimated from figures in two trials [26, 27], or one trial that used primarily meditation practices [40] that have overlapping features with relaxation training or other counter-arousal methods of CBT-I, results did not substantially change [MD’s: -4.4 (-5.4, -3.5) points; I^2^ = 63%; and − 4.4 (-5.4, -3.4) points, I^2^ = 66%, respectively). Using the SMD approach in sensitivity analyses, the effect size (95% CI) of CBT-I on ISI at end-of-intervention, compared with controls, was: -0.9 (-1.1, -0.7), statistically large-to-very-large that on average exceeded the SMD threshold of ≥ 0.50, suggesting clinical meaningfulness.

### Effect of CBT-I on the ISI at follow up (1–3, 6, and 12-Months)

Of the 16 RCTs that assessed ISI, a minority (< 50%) had follow up beyond one month. At 1–3 and 6 months postintervention, the ISI reduction remained lower among CBT-I compared to control participants, with possibly smaller magnitude in reduction compared to end-of-intervention: MD’s -3.3 (-4.3, -2.2) (7 RCTs; *N* = 758; I^2^ = 59%) and − 3.5 (-4.7, -2.3) (7 RCT; *N* = 576; I^2^ = 59%) points, respectively, that did not reach the MCID to suggest that many participants derived clinically-important benefit, but could have been greater than half of the MIC (95% CI’s crossed 3 points), suggesting that possibly an appreciable number of participants derived clinically-important benefits; there was no statistically-significant or clinically-important effects between groups at 12-month follow-up [MD: -2.9 (-5.8, + 0.04) points] (3 RCTs; *N* = 308; I^2^ = 83%; unreliable). Sensitivity analyses of between-group SMD’s (95% CI) at end-of intervention, 1–3, and 6-month follow-up were: -0.91 (-1.09, -0.72); -0.71 (-1.0, -0.40); and − 0.80 (-1.2, -0.40), respectively, statistically large-to-very-large effects, suggestive of clinical meaningfulness (i.e., SMD ≥ 0.50).

### Effects of CBT-I on sleep parameters, fatigue, HRQL (2⁰ Outcomes) at end-of-intervention

At baseline, there were no differences in sleep behaviors/parameters, fatigue, or HRQL between groups. At end-of-intervention, compared with controls, participants in the CBT-I group reported improved sleep diary behaviors/parameters [MD’s (95% CI’s)]: sleep latency [-11 (-15, -8) *minutes*] (12 RCTs; *N* = 936; I^2^ = 38%) (low-certainty); WASO [-14.7 (-20.8, -8.5) *minutes*] (12 RCTs, *N* = 937; I^2^ = 65%) (low-certainty), and efficiency + 7.0 (+ 5.2, + 8.7) *percent* (13 RCTs; *N* = 994; I^2^ = 38%) (very-low-certainty). None of these parameters reached the MCID thresholds (including 95% CI’s) [37], with sleep latency, WASO, and sleep efficiency exceeding half of the MIC, suggesting that an appreciable number, not many, of participants derived clinically-important benefits [36] (low-to-very-low certainty) (Table 3). Sleep duration was not statistically different between groups at end-of-intervention: +4.5 (-6.9, + 15.9) *minutes* (12 RCTs; *N* = 974; I^2^ = 48%) (very-low-certainty). In sensitivity analyses, the SMD’s (95% CI’s) for subjective/sleep diary sleep latency, WASO, and sleep latency were: -0.56 (-0.73, -0.39); -0.53 (-0.73, -0.34); and + 0.67 (+ 0.52, + 0.83), all greater than the ≥ 0.50 threshold suggestive of clinical meaningfulness.

A minority (6, 32%) of trials assessed objective sleep behaviors/parameters using actigraphy or sleep study. Compared to sleep diary, the magnitude of improvements detected by actigraphy at end-of-intervention were generally lower [sleep latency: -4.0 (-6.4, -1.6) *minutes* (4 RCTs; *N* = 331; I^2^ = 0%); WASO: -9.0 (-17.7, -0.3) *minutes* (4 RCTs; *N* = 335; I^2^ = 79%; unreliable)] that again, did not reach the MCID thresholds (including 95% CI’s) [37] but reached close to half of the MIC (i.e., 5-minute and 10-minute reductions), suggesting that potentially an appreciable number of participants derived clinically-important benefits (unreliable evidence, due to substantial heterogeneity I^2^ > 70%, small number of < 7 RCTs, and wide 95% CI’s). There were no statistically-significant differences between groups in end-of-intervention sleep duration [-0.45 (-24.1, + 23.2) minutes] (4 RCTs; *N* = 370; I^2^ = 83%; unreliable) or sleep efficiency [+ 1.5 (-1.0, + 3.9) percent] (5 RCTs; *N* = 395; I^2^ = 79%; unreliable). Two trials (*N* = 121; I^2^ = 0%) assessed sleep parameters using sleep study, with none of the sleep behavior / parameters having differences between groups at end-of-intervention.

Moreover, compared with controls, there was a statistical reduction in fatigue among CBT-I participants at end-of-intervention, with SMD (95% CI) -0.29 (-0.4; -0.15) (11 RCTs; *N* = 831; I^2^ = 0%) (low-certainty) (Fig. 3A); similarly, HRQL was statistically improved [SMD (95% CI)]: +0.2 (+ 0.04, + 0.36) (10 RCTs; *N* = 626; I^2^ = 0%) (very-low-certainty) (Fig. 3B). However, the magnitude of these statistical improvements were small and did not reach the SMD threshold ≥ 0.50 to suggest clinical meaningfulness [37] (Table 3).

**Fig. 3A:**
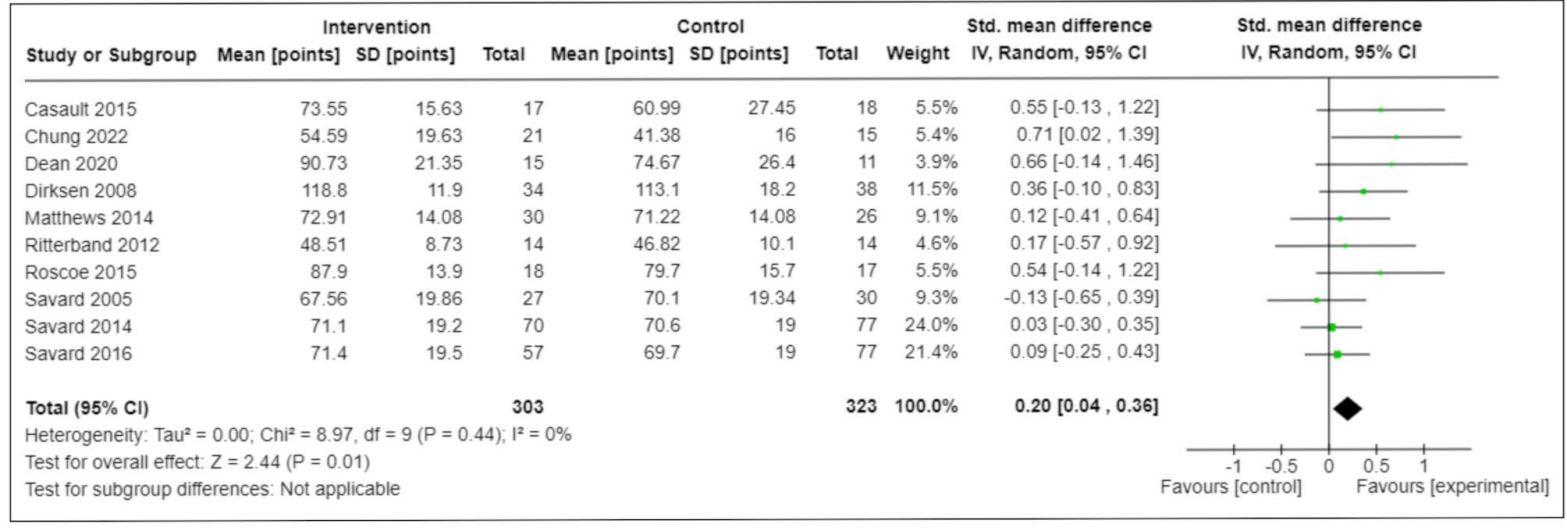
Between-Group Differences in End-of-Intervention Fatigue (Secondary Outcome) CI = confidence interval; ISI = Insomnia Severity Index; SD = standard deviation

**Fig. 3B:**
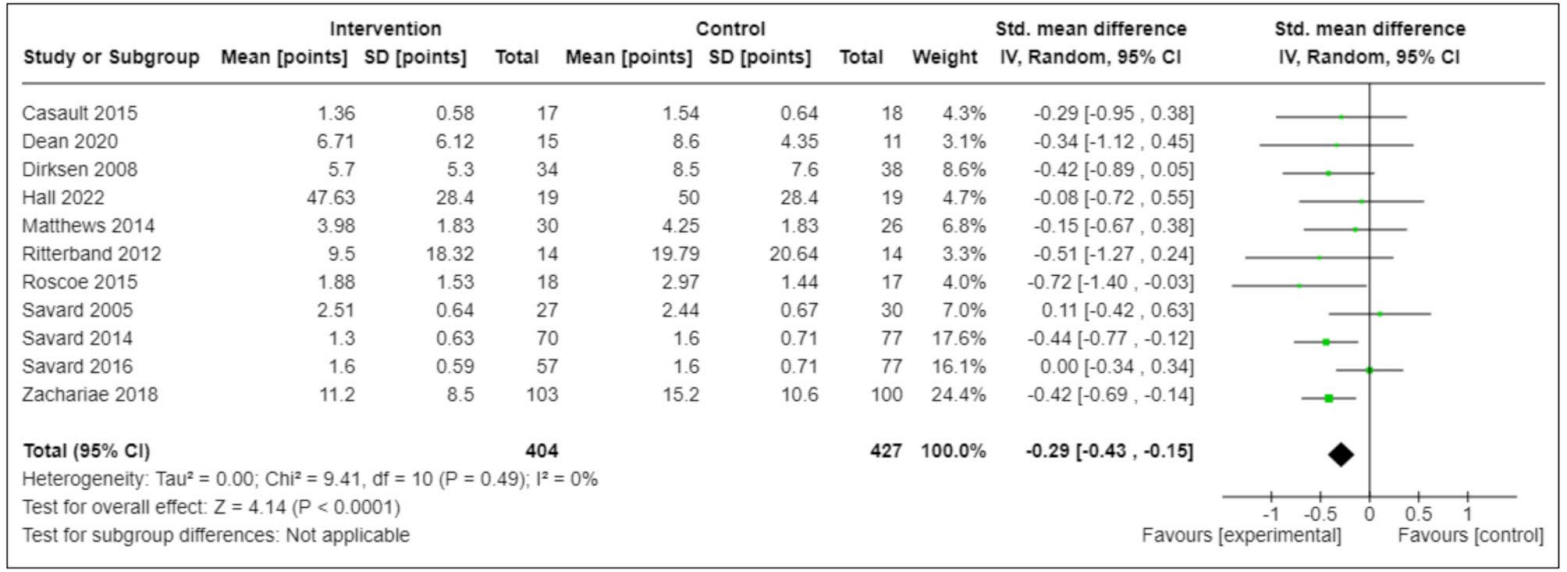
Between-Group Differences in End-of-Intervention HRQL^*a*^ (Secondary Outcome) ^a^Large variations in means and SD’s due to differences in HRQL measures used CI = confidence interval; ISI = Insomnia Severity Index; HRQL = health-related quality of life; SD = standard deviation

In all, the strength of the evidence for primary and all secondary outcomes at end-of-intervention were low or very-low, due to heterogeneity, performance, reporting, and/or publication bias, with none of the outcomes reaching their respective MCID thresholds (Table 3). The magnitudes of treatment effects on ISI, sleep diary/subjective sleep latency, WASO, and sleep efficiency reached close to or exceeded half of the MIC’s, suggesting that an appreciable number, not many, of participants derived clinically-important benefits at end-of-intervention.

## Discussion

This systematic review and meta-analysis found with low to very-low certainty evidence that CBT-I, compared with controls, statistically improved the primary outcome ISI and secondary outcomes patient-reported sleep latency, WASO, sleep efficiency, fatigue, and HRQL that on average, did not reach their respective MCID thresholds to indicate that many patients derived clinically-important benefits at end-of-intervention, with unreliable evidence on objective actigraphy sleep parameters. In addition, a minority (< 50%) of studies assessed these outcomes beyond end-of-intervention. These results have important implications for additional and/or alternative strategies to improve insomnia for the many cancer survivors who do not respond to traditional, first-line CBT-I treatment.

An important distinction of our meta-analysis from priors is the use of between-group MD and MCID thresholds instead of within-group changes or statistical SMD’s to interpret results, as recommended to evaluate intervention effects [42] and is a limitation of existing literature as raised by the ACP [36]. As such, we included only RCTs in which appropriate controls (e.g., wait-list, sleep hygiene education only) were used to better evaluate treatment effects. Most recently, Squires and colleagues updated an older meta-analysis and found that, across 15 trials involving 1,461 participants, CBT-I statistically reduced ISI from pre-to-postintervention by an average of -7.8 points (SMD 0.78) that exceeded the MIC threshold [15]. However, that meta-analysis included four trials that we excluded based on our pre-registered protocol to examine between-group effects: one due to a cross-over design in which participants in the treatment and control groups received the intervention before outcome assessments [43], two due to non-inferiority designs that compared home-based exercise [44] or Tai-Chi [45] with CBT-I, both of which reported significant improvements in insomnia among the comparator group, and another due to the absence of peer-review on grey literature involving a low-intensity CBT-I intervention [46] that may diminish true treatment effects [47]. Despite these differences, secondary analyses from that meta-analysis (based on our review of their registered protocol) also identified that participants in the CBT-I group, compared with the comparator group, derived an average ISI reduction of -4.3 points at end-of-intervention (results we manually calculated) that is similar to our findings (those in the comparator group also improved by -3.5 points). Also like our study, the effects of CBT-I appeared to numerically decrease over time, were generally smaller for objective compared to subjective sleep behaviors/parameters, and small or very-small on downstream patient-centered outcomes fatigue and HRQL [15].

Our meta-analysis adds to the existing literature on CBT-I among cancer survivors in that our expanded and updated search includes two additional years of literature and three additional large databases omitted in the previous review [15]. As a result, we identified 3,476 more records and screened 1,646 more unique studies, and – because we excluded 4 trials involving 183 participants included in that review – 8 new RCTs and 525 additional participants [15]. In addition, we detected potential publication bias, possibly due to higher sensitivity of the Egger’s compared to Begg’s test to detect publication bias, as also detected by the ACP guideline [36]. Moreover, we rated the strength of evidence using GRADE, not performed in prior meta-analyses [14, 15], to facilitate interpretation and decision-making.

Interpretation of intervention effects is recommended to be informed by the between-group MCIDs instead of statistical approaches such as the SMD unless necessary. We used a single primary outcome measure (i.e., ISI) to enhance interpretation and anchored results interpretation on a 6-point MCID threshold established among individuals with chronic insomnia [24, 33] and endorsed by the ACP guideline [36]. In doing so, we provided evidence that an appreciable number, not many, of cancer survivors derived clinically-important benefits with traditional CBT-I. While these results are limited by the absence of an established or well-accepted MCID threshold in cancer and our inability to conduct participant-level analyses, it is highly likely that many (i.e., > 50%) of the participants did not reach the MCID threshold, as also reported in a 2023 pragmatic RCT evaluating sleep restriction therapy among individuals with chronic insomnia [48]. The importance of analyses to examine responders and non-responders has been raised by the ACP [36], with recent calls in precision sleep medicine to adapt CBT-I to better address cancer-specific challenges, such as fear of cancer recurrence and progression [3]. These findings have implications in the design of future interventions, including CBT for cancer-related insomnia, and methodological considerations to better examine treatment effects.

Moreover, we identified moderate-to-substantial clinical and methodological heterogeneity among RCTs evaluating CBT-I among cancer survivors, notably related to time since cancer diagnosis that ranged 2 months to 8 years and with CBT-I interventions that ranged 1 to 16 total hours, highlighting a limitation of existing literature in this population to more concretely define the population with regards to timing along the cancer life course and more uniform interventions to reduce heterogeneity and improve the certainty of evidence. Further, we identified an overwhelming number of > 90% of participants enrolled to date being women, predominantly survivors of breast cancer, which highlight a need to include men and survivors of other cancer types with insomnia.

The strengths of our systematic review and meta-analysis include: (1) a more comprehensive and updated search strategy that resulted in a substantial number of 8 new RCTs and 525 more participants included; (2) conduct following standard Cochrane Handbook recommendation for systematic reviews of intervention effects, including between-group comparison with appropriate controls; (3) use of a directed acyclic graph to outline potential mechanisms; (4) thorough evaluation of CBT-I components, cancer-related characteristics, subgroup, and sensitivity analyses; (5) use of guideline-endorsed MCID thresholds to interpret results; and (6) GRADE ratings to inform decision-making and future studies.

Limitations include an approximately 2-year lag between search completion and analyses; however this 2-year lag is similar to prior systematic reviews and meta-analyses [14, 15], with a substantial greater number of studies identified and full-length manuscripts reviewed. Additional limitations include absence of individual participant data analyses, limited availability of RCTs that reported participant-level changes, and small number of adequately-powered phase III RCTs to determine treatment effect. Further, the very low inclusion of men (6%) and low inclusion of survivors of cancers other than breast limit the generalizability of findings to male cancer survivors and/or those facing sleep related challenges not due to hormonal or other factors related to female breast cancer survivors.

Future RCTs can enroll male cancer survivors and/or evaluate CBT-I among non-breast cancer survivors to enhance clinical diversity and generalizability. In addition, evaluation of psychometric properties, including MCID thresholds, for the ISI and other sleep measures in cancer is needed to advance the field. Future work can also update this systematic review and meta-analysis, recognizing the inherent time lag incurred in a rigorously conducted study that includes thorough search, review of results, consensus, data extraction, rating of bias, meta-analysis, and grading of the strength of evidence.

We conclude with low-to-very low certainty evidence that among cancer survivors, CBT-I, compared with controls, improved ISI and subjective sleep diary parameters that on average, did not reach the respective MCID but reached half of the MIC thresholds, suggesting that an appreciable number, not many, of participants derived clinically-important benefits. Future work can conduct methodologically rigorous RCTs to evaluate CBT-I or other interventions to improve insomnia among cancer survivors, enroll men and survivors of non-breast cancer, establish MCID thresholds in cancer, and evaluate intervention effects by examining participant-level changes.

## Electronic supplementary material

Below is the link to the electronic supplementary material.

Supplementary Material 1

## Data Availability

Data is provided within the manuscript and supplementary information files.

## Abbreviations

AASM: American Academy of Sleep Medicine
ACP: American College of Physicians
BBT-CI: Brief Behavioral Therapy for Cancer-Related Insomnia
BBT-I: Brief Behavioral Therapy for Insomnia
CBT-I: Cognitive Behavioral Therapy for Insomnia
CINAHL: Cumulative Index of Nursing and Allied Health
DSM-IV: Diagnostic & Statistical Manual of Mental Disorders, 4th edition
HRQL: Health-Related Quality of Life
ICSD: International Classification of Sleep Disorders
IIS: Insomnia Interview Schedule
ISI: Insomnia Severity Index
MCID: Minimal Clinically Important Difference
MD: Mean Difference
MIC: minimal important change
NR: not reported
PEDro: Physiotherapy Evidence Database
PSQI: Pittsburgh Sleep Quality Index
RCT: Randomized Controlled Trial
RoB: Risk of Bias
SDEF: Sleep Disruption Evaluation Form
SE: Standard Error
SLE: sleep efficiency
SMD: Standardized Mean Difference
SOL: Sleep Onset Latency
WASO: Wake After Sleep Onset
