# Supplementary material for: Effects of cognitive-behavioral therapy for insomnia compared with controls among cancer survivors: a systematic review and meta-analysis of randomized trials
Source: BMC Cancer. 2025 May 14;25:871. doi: 10.1186/s12885-025-14192-y (PMC12079849; doi:10.1186/s12885-025-14192-y)
Supplement: Supplementary file 1 — Supplementary Material 1 [file 12885_2025_14192_MOESM1_ESM.docx]

**Online Supplemental/Additional Data**

Effects of Cognitive-Behavioral Therapy for Insomnia Compared with Controls Among Cancer Survivors: A Systematic Review and Meta-analysis of Randomized Trials

Joshua T. Cooper, BS; Ellie Svobada, MLIS, AHIP; Allan V. Prochazka, MD, MSc; Duc M. Ha, MD, MAS

**E-Table 1:** Search Strategy*^*^*

| Ovid MEDLINE | | |
| --- | --- | --- |
| Search sequence | Search terms | Results |
| 1 | ((randomized controlled trial or controlled clinical trial).pt. or randomized.ab. or placebo.ab. or randomly.ab. or trial.ab. or groups.ab. or clinical trials as topic.sh.) not (exp animals/ not humans.sh.) | 3,054,436 |
| 2 | (Cancer* or Neoplas* or Tumor* or tumour* or Lymphoma or Leukemia or Malignan* or carcinoma*).tw,kf. or exp Neoplasms/ or exp "Cancer Survivors" | 4,876,027 |
| 3 | (((Cognitive or Behavio*) adj3 therap*) or BBTI or Psychotherap* or stimulus control or sleep restriction therap* or relaxation or biofeedback or paradoxical intervention* or intensive sleep retraining or mindfulness or meditation or psychology or counsel* or psychosocial or sleep medicine specialty or CBT).tw,kf. or exp "Cognitive Behavioral Therapy"/ | 528,551 |
| 4 | (Sleep* or Insomnia* or hyposomnia or agrypnia or polysomnograph* or actigraph*).tw,kf. or exp "Sleep Initiation and Maintenance Disorders"/ or exp "Sleep Wake Disorders"/ or exp "Sleep Hygiene"/ | 245,963 |
| 5 | 1 and 2 and 3 and 4 | 522 |
| 6 | limit 5 to English language | 515 |

*^*^Displays search strategy run in Ovid Medline only. Each search number displays the search criteria that were used in order.*

**E-Table 2:** Definitions of CBT-I, BBT-I, and Individual Components [1]

| Intervention | Description |
| --- | --- |
| Multi-component Interventions | |
| CBT-I | CBT-I combines one or more of the cognitive therapy strategies with education about sleep regulation plus stimulus control instructions and sleep restriction therapy. CBT-I also often includes sleep hygiene education, relaxation training, and other counter-arousal methods. Treatment progresses using information typically gathered with sleep diaries completed by the patient throughout the course of treatment (typically 4-8 sessions) |
| BBT-I | BBT-I includes abbreviated versions of CBT-I (typically 1-4 sessions) emphasizing the behavioral components. BBT-I typically consists of education about sleep regulation, factors that influence sleep, and behaviors that promote or interfere with sleep, along with a tailored behavioral prescription based on stimulus control and sleep restriction therapy and on information typically derived from pretreatment sleep diary. Some therapies include brief relaxation or cognitive therapy elements. |
| Specific Components | |
| Sleep hygiene | A set of general recommendations about lifestyle (e.g., diet, exercise, substance use) and environmental factors (e.g., light, noise, temperature) that may promote or interfere with sleep. Sleep hygiene may include some education about what constitutes “normal” sleep and changes in sleep patterns with aging. |
| Stimulus control | A set of instructions designed to (1) extinguish the association between the bed/bedroom and wakefulness to restore the association of bed/bedroom with sleep; and (2) establish a consistent wake-time. Stimulus control instructions are: (a) go to bed only when sleepy; (b) get out of bed when unable to sleep; (c) use the bed/bedroom for sleep and sex only (no reading, watching TV, etc. in bed); (d) wake up the same time every morning; (e) refrain from daytime napping. |
| Sleep restriction therapy | A method designed to enhance sleep drive and consolidate sleep by limiting time in bed equal to the patient’s sleep duration, typically estimated from daily diaries. Time in bed is initially limited to the average sleep duration, and subsequently increased or decreased based on sleep efficiency thresholds, until sufficient sleep duration and overall sleep satisfaction is achieved. |
| Relaxation therapy | Structured exercises designed to reduce somatic tension (e.g., abdominal breathing, progressive muscle relaxation; autogenic training) and cognitive arousal (e.g., guided imagery training; meditation) that may perpetuate sleep problems. |
| Cognitive therapy | A set of strategies including structured psychoeducation, Socratic questioning, use of thought records, and behavioral experiments designed to identify and modify unhelpful beliefs about sleep that may support sleep-disruptive habits and/or raise performance anxiety about sleeping. |

BBT-I = Brief-Behavioral Therapy for Insomnia; CBT-I = Cognitive-Behavioral Therapy for Insomnia

**E-Table 3:** Summary of Directionality of Fatigue and HRQL Measures

| First author,  year | 1. Fatigue | | | 1. HRQL | | |
| --- | --- | --- | --- | --- | --- | --- |
|  | Measure | Score range | Direction*^a^* | Measure | Score range | Direction*^a^* |
| Casault,  2015 [2] | MFI | 1-5 | Higher | EORTC C30  global health | 0-100 | Higher |
| Chung,  2022 [3] | POMS-SF | 0-20 | Higher | SF-36 | 0-100 | Higher |
| Dean,  2020 [4] | POMSF/I | 0-28 | Higher | FACT-L | 0-136 | Higher |
| Dirksen,  2008 [5] | FSI | 0-130 | Higher | FACT –  Breast | 0-148 | Higher |
| Espie,  2010 [6] | FSI | 0-130 | Higher | FACT –  General | 0-108 | Higher |
| Matthews,  2014 [7] | PFS | 0-10 | Higher | EORTC C30  global health | 0-100 | Higher |
| Ritterband,  2012 [8] | MFSI-SF | 24-96 | Higher | SF-12 | 0-100 | Higher |
| Roscoe,  2015 [9] | BFI | 0-10 | Higher | FACT –  General | 0-108 | Higher |
| Savard,  2005 [10] | MFI | 1-5 | Higher | EORTC C30  global health | 0-100 | Higher |
| Savard,  2014 [11] | MFI | 1-5 | Higher | EORTC C30  global health | 0-100 | Higher |
| Savard,  2016 [12] | FACIT-F | 0-52 | Lower*^*^* | EORTC C30  global health | 0-100 | Higher |

*^a^Higher scores correlate with greater fatigue or better HRQL.*

*^b^Lower scores correlate with greater fatigue; means re-calculated to align directionality by subtracting the maximum score (52 points) from the mean; the standard deviation was not modified [13].*

BFI = Brief Fatigue Inventory; EORTC C30 = European Organization for Research and Treatment of Cancer Core 30; FACIT-F = Functional Assessment of Chronic Illness Therapy – Fatigue subscale; FACT = Functional Assessment of Cancer Therapy; FSI = Fatigue Symptom Inventory; MFI/SF = Multidimensional Fatigue Inventory/Short-Form; PFS = Piper Fatigue Scale: POMSF/I = Profile of Mood States – Fatigue/Inertia subscale; SF-12/36 = Short Form Health Survey 12/36-items

**E-Table 4:** CBT-I Components Used in RCTs

| First author,  year | Sleep  hygiene | Stimulus  control | Sleep  restriction | Cognitive  therapy | Relaxation  training | Total  components | BBT-I | CBT-I |
| --- | --- | --- | --- | --- | --- | --- | --- | --- |
| Palesh,  2018 [14] | ✓ | ✓ | ✓ |  |  | 3 | ✓ |  |
| Savard,  2016 [12] | ✓ | ✓ | ✓ | ✓ |  | 4 |  | ✓ |
| Savard,  2014 [11] | ✓ | ✓ | ✓ | ✓ |  | 4 |  | ✓ |
| Casault,  2015 [2] | ✓ | ✓ | ✓ | ✓ |  | 4 |  | ✓ |
| Savard,  2005 [10] | ✓ | ✓ | ✓ | ✓ |  | 4 |  | ✓ |
| Dirksen,  2008 [5] | ✓ | ✓ | ✓ |  |  | 3 |  | ✓ |
| Hall,  2022 [15] | ✓ | ✓ |  | ✓ | ✓ | 4 |  | ✓ |
| Zhao,  2020 [16] | ✓ |  |  | ✓ | ✓ | 3 |  |  |
| Zachariae,  2018 [17] | ✓ | ✓ | ✓ | ✓ |  | 4 |  | ✓ |
| Ritterband,  2012 [8] | ✓ | ✓ | ✓ | ✓ |  | 4 |  | ✓ |
| Roscoe,  2015 [9] | ✓ | ✓ | ✓ | ✓ |  | 4 |  | ✓ |
| Gonzalez,  2022 [18] | ✓ | ✓ | ✓ | ✓ |  | 4 |  | ✓ |
| Espie,  2010 [6] | ✓ | ✓ | ✓ | ✓ |  | 4 |  | ✓ |
| Matthews,  2014 [7] | ✓ | ✓ | ✓ | ✓ |  | 4 |  | ✓ |
| Palesh,  2020 [19] | ✓ | ✓ | ✓ |  |  | 3 |  | ✓ |
| Dean,  2020 [4] | ✓ | ✓ | ✓ |  |  | 3 | ✓ |  |
| Padron,  2019 [20] | ✓ | ✓ | ✓ | ✓ | ✓ | 5 |  | ✓ |
| Zhang,  2019 [21] |  |  |  |  | ✓ | 1 | N/A*^*^* | N/A*^*^* |
| Chung,  2022 [3] | ✓ | ✓ | ✓ | ✓ | ✓ | 5 |  | ✓ |

*^*^One RCT used a group-based meditation approach that meets the AASM definition of “relaxation training” or “other counter-arousal methods” but not traditionally considered CBT-I. Excluded in sensitivity analyses.*

AASM = American Academy of Sleep Medicine; BBT-I = Brief-Behavioral Therapy for Insomnia; CBT-I = Cognitive-Behavioral Therapy for Insomnia; RCT = randomized controlled trial

**E-Figure 1:** Directed Acyclic Graph of Hypothesized Mechanisms and CBT-I Treatment Effects*^*^*


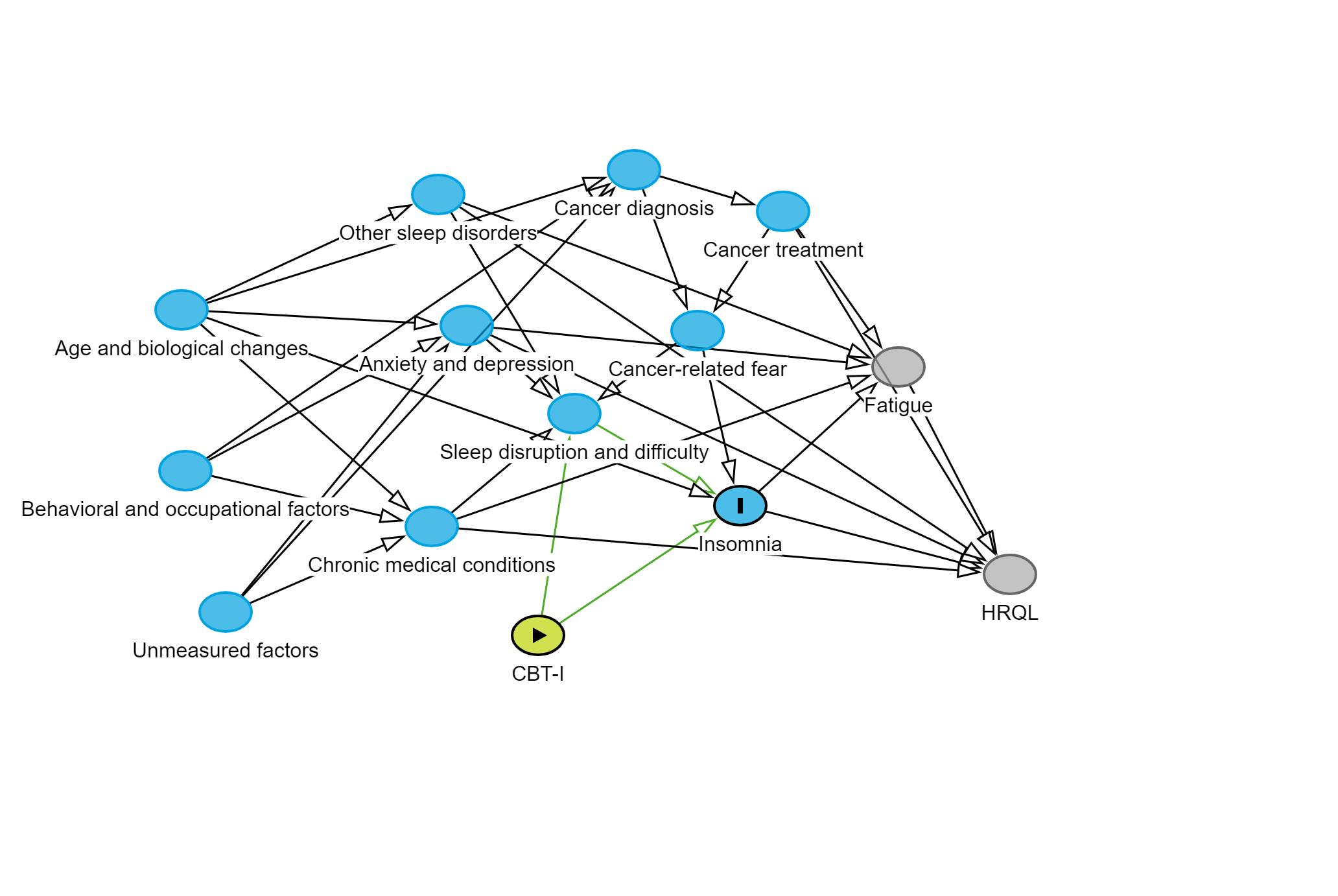


*^*^Following cancer diagnosis and treatment, cancer survivors experience sleep disruptions and difficulties related to cancer and its treatments, cancer-related fear/worries, and along with age and biological changes, behavioral and occupational factors, comorbid conditions including anxiety and depression, can lead to clinically-significant insomnia. Cognitive-behavioral therapy for insomnia improves sleep behaviors, cognitive restructuring, and sleep difficulties to control insomnia, which in turn, improves fatigue and enhances HRQL.*

CBT-I = cognitive-behavioral therapy for insomnia; HRQL = health-related quality life

**E-Figure 2:** Risk of Bias Assessments


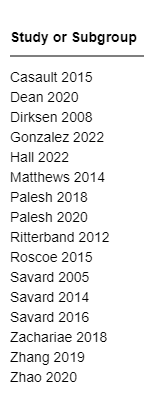

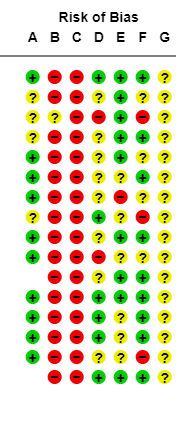


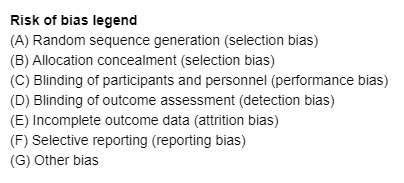


(+)/green indicates low concern of bias.

(?)/yellow indicates uncertain risk of bias.

(-)/red indicates significant risk of bias.

**E-Figure 3:** Funnel Plots of ISI and Sleep Parameters*^*^*


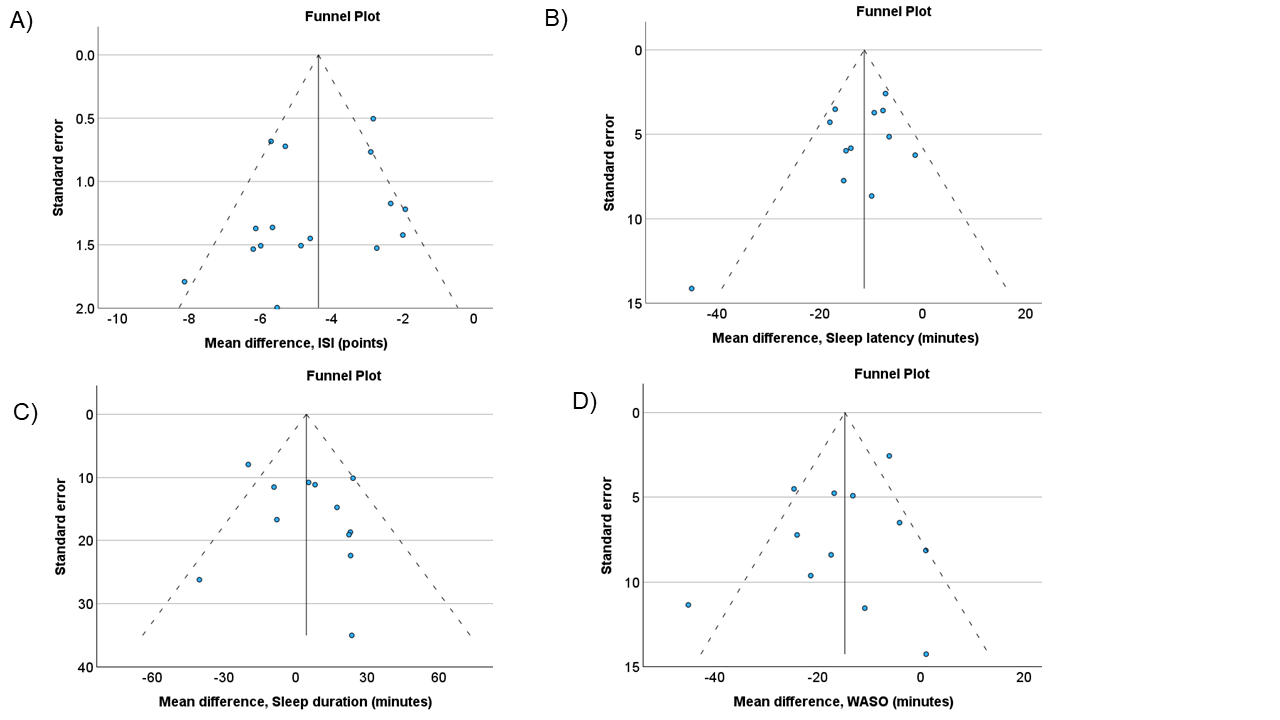


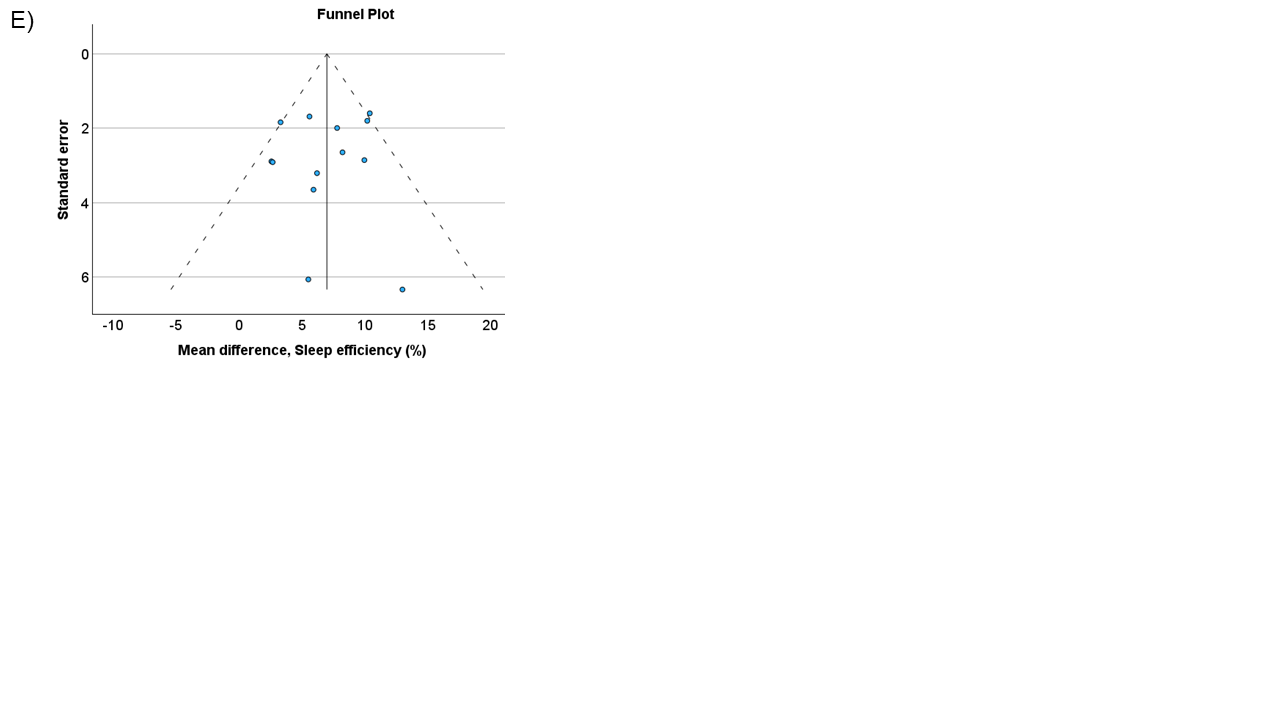


*^*^Egger’s test p <0.05 for funnel plots suggests the presence of publication bias: A)* p=0.045; B) p=0.28; C) p=0.80; D) p=0.17; E) p=0.01

ISI = Insomnia Severity Index; WASO = wake after sleep onset

**References**

[1] J. D. Edinger *et al.*, "Behavioral and psychological treatments for chronic insomnia disorder in adults: an American Academy of Sleep Medicine clinical practice guideline," *Journal of Clinical Sleep Medicine,* vol. 17, no. 2, pp. 255-262, 2021.

[2] L. Casault, J. Savard, H. Ivers, and M. H. Savard, "A randomized-controlled trial of an early minimal cognitive-behavioural therapy for insomnia comorbid with cancer," *Behaviour Research & Therapy,* vol. 67, pp. 45-54, 2015, doi: <https://dx.doi.org/10.1016/j.brat.2015.02.003>.

[3] K. M. Chung *et al.*, "A Pilot Study Testing the Efficacy of dCBT in Patients With Cancer Experiencing Sleep Problems," *Frontiers in psychology,* vol. 13, p. 699168, 2022, doi: <https://dx.doi.org/10.3389/fpsyg.2022.699168>.

[4] G. E. Dean *et al.*, "Nurse-Delivered Brief Behavioral Treatment for Insomnia in Lung Cancer Survivors: A Pilot RCT," *Behavioral sleep medicine,* vol. 18, no. 6, pp. 774-786, 2020, doi: <https://dx.doi.org/10.1080/15402002.2019.1685523>.

[5] S. R. Dirksen and D. R. Epstein, "Efficacy of an insomnia intervention on fatigue, mood and quality of life in breast cancer survivors," *Journal of advanced nursing,* vol. 61, no. 6, pp. 664-75, 2008, doi: <https://dx.doi.org/10.1111/j.1365-2648.2007.04560.x>.

[6] Espie, "Randomized controlled clinical effectiveness trial of cognitive behavior therapy compared with treatment as usual for persistent insomnia in patients with cancer (Journal of Clinical Oncology (2008) 26, (4651-4658))," *Journal of Clinical Oncology,* vol. 28, no. 19, p. 3205, 2010, doi: 10.1200/JCO.2010.30.8577.

[7] E. E. Matthews *et al.*, "Cognitive behavioral therapy for insomnia outcomes in women after primary breast cancer treatment: a randomized, controlled trial," *Oncology nursing forum,* vol. 41, no. 3, pp. 241-53, 2014, doi: <https://dx.doi.org/10.1188/14.ONF.41-03AP>.

[8] L. M. Ritterband, E. T. Bailey, F. P. Thorndike, H. R. Lord, L. Farrell-Carnahan, and L. D. Baum, "Initial evaluation of an Internet intervention to improve the sleep of cancer survivors with insomnia," *Psycho-oncology,* vol. 21, no. 7, pp. 695-705, 2012, doi: <https://dx.doi.org/10.1002/pon.1969>.

[9] J. A. Roscoe *et al.*, "Randomized placebo-controlled trial of cognitive behavioral therapy and armodafinil for insomnia after cancer treatment," *Journal of Clinical Oncology,* vol. 33, no. 2, pp. 165-71, 2015, doi: <https://dx.doi.org/10.1200/JCO.2014.57.6769>.

[10] J. Savard, S. Simard, H. Ivers, and C. M. Morin, "Randomized study on the efficacy of cognitive-behavioral therapy for insomnia secondary to breast cancer, part I: Sleep and psychological effects," *Journal of Clinical Oncology,* vol. 23, no. 25, pp. 6083-96, 2005. [Online]. Available: <https://ovidsp.ovid.com/ovidweb.cgi?T=JS&CSC=Y&NEWS=N&PAGE=fulltext&D=med6&AN=16135475>.

[11] J. Savard, H. Ivers, M. H. Savard, and C. M. Morin, "Is a video-based cognitive behavioral therapy for insomnia as efficacious as a professionally administered treatment in breast cancer? Results of a randomized controlled trial," *Sleep,* vol. 37, no. 8, pp. 1305-14, 2014, doi: <https://dx.doi.org/10.5665/sleep.3918>.

[12] J. Savard, H. Ivers, M.-H. Savard, and C. M. Morin, "Long-term effects of two formats of cognitive behavioral therapy for insomnia comorbid with breast cancer," *Sleep: Journal of Sleep and Sleep Disorders Research,* vol. 39, no. 4, pp. 813-823, 2016, doi: <https://dx.doi.org/10.5665/sleep.5634>.

[13] L. T. Higgins JPT, Deeks JJ, "Chapter 6: Choosing effect measures and computing estimates of effect," in *Cochrane Handbook for Systematic Reviews of Interventions*, T. J. Higgins JPT, Chandler J, Cumpston M, Li T, Page MJ, Welch VA Ed. <www.training.cochrane.org/handbook>, 2023, ch. 6.

[14] O. Palesh *et al.*, "Feasibility and acceptability of brief behavioral therapy for cancer-related insomnia: effects on insomnia and circadian rhythm during chemotherapy: a phase II randomised multicentre controlled trial," *British journal of cancer,* vol. 119, no. 3, pp. 274-281, 2018, doi: <https://dx.doi.org/10.1038/s41416-018-0154-2>.

[15] D. L. Hall *et al.*, "The Survivorship Sleep Program (SSP): A synchronous, virtual cognitive behavioral therapy for insomnia pilot program among cancer survivors," *Cancer,* vol. 128, no. 7, pp. 1532-1544, 2022, doi: <https://dx.doi.org/10.1002/cncr.34066>.

[16] Y. Zhao *et al.*, "Effects of mindfulness-based cognitive therapy on breast cancer survivors with insomnia: A randomised controlled trial," *European journal of cancer care,* vol. 29, no. 5, p. e13259, 2020, doi: <https://dx.doi.org/10.1111/ecc.13259>.

[17] R. Zachariae *et al.*, "Internet-Delivered Cognitive-Behavioral Therapy for Insomnia in Breast Cancer Survivors: A Randomized Controlled Trial," *Journal of the National Cancer Institute,* vol. 110, no. 8, pp. 880-887, 2018, doi: <https://dx.doi.org/10.1093/jnci/djx293>.

[18] B. D. Gonzalez *et al.*, "PILOT RANDOMIZED CONTROLLED TRIAL OF SPANISH EHEALTH COGNITIVE-BEHAVIORAL THERAPY FOR INSOMNIA IN BREAST CANCER SURVIVORS," *Annals of Behavioral Medicine,* vol. 56, no. SUPP 1, pp. S518-S518, 2022. [Online]. Available: <Go to ISI>://WOS:000788118601457.

[19] O. Palesh *et al.*, "A novel approach to management of sleep-associated problems in patients with breast cancer (MOSAIC) during chemotherapy: A pilot study," *Sleep: Journal of Sleep and Sleep Disorders Research,* vol. 43, no. 10, pp. 1-11, 2020, doi: <https://dx.doi.org/10.1093/sleep/zsaa070>.

[20] A. Padron *et al.*, "Longitudinal cognitive behavioral effects on subjective sleep outcomes in women with gynecologic malignancies and insomnia," *Supportive Care in Cancer,* vol. 27, no. 1, pp. S204-S205, 2019, doi: 10.1007/s00520-019-04813-1.

[21] H. Zhang, Y. Li, M. Li, and X. Chen, "A randomized controlled trial of mindfulness-based stress reduction for insomnia secondary to cervical cancer: Sleep effects," *Applied Nursing Research,* vol. 48, pp. 52-57, 2019, doi: <https://dx.doi.org/10.1016/j.apnr.2019.05.016>.
